# Exposure to low environmental copper concentrations does not affect survival and development in Atlantic cod (*Gadus morhua*) early life stages

**DOI:** 10.1016/j.toxrep.2021.11.012

**Published:** 2021-11-24

**Authors:** Julia Farkas, Linn H. Svendheim, Tjalling Jager, Tomasz M. Ciesielski, Trond Nordtug, Bjarne Kvæstad, Bjørn H. Hansen, Torstein Kristensen, Dag Altin, Pål A. Olsvik

**Affiliations:** a SINTEF Ocean, Environment and New Resources, Trondheim, Norway; b Nord University, Faculty of Biosciences and Aquaculture, Bodø, Norway; c DEBtox Research, Stevensweert, the Netherlands; d Department of Biology, Norwegian University of Science and Technology, Trondheim, Norway; e BioTrix, Trondheim, Norway

**Keywords:** Copper, Marine environment, Low concentrations, Fish early life stages, DEB, Atlantic cod

## Abstract

In this study we investigated potential impacts of Cu exposure at low, environmentally relevant, concentrations on early live stages of Atlantic cod (*Gadus morhua*). Cod embryos and larvae were exposed to 0.5 μg/L (low), 2 μg/L (medium), and 6 μg/L (high) Cu from 4 to 17 days post fertilisation (dpf). Hatching success, mortality, oxygen consumption, biometric traits, and malformations were determined. A dynamic energy budget (DEB) model was applied to identify potential impacts on bioenergetics. A positive correlation was found between Cu exposure concentrations and Cu body burden in eggs, but not in larvae. The tested concentrations did not increase mortality in neither embryos nor larvae, or larvae deformations. Further, the DEB model did not indicate effects of the tested Cu concentrations.

## Highlights

• Early life stages of cod were exposed to environmentally relevant Cu concentrations.
• Copper significantly accumulated in eggs but not in larvae.
• Exposure to the tested Cu concentrations did not increase mortality or deformations.
• Low Cu exposure did not affect development and growth of cod early life stages.

## Graphical abstract

**Figure:**
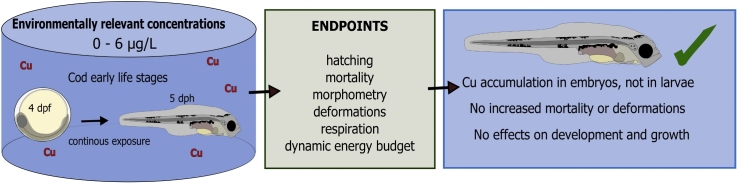

## 1 Introduction

As most heavy metals, Cu is a natural constituent in the marine environment, with background concentrations in estuarine and coastal waters ranging from 0.02 to 3 μg/L [[1], [2], [3]]. However, human activities can significantly increase environmental Cu concentrations, with some of the major anthropogenic sources to the marine environment being industrial effluents, municipal wastewater, biocides such as marine antifouling coatings and paints and mining activities, causing an increasing trend in sediment copper concentrations in exposed areas [[3], [4], [5], [6]].

For several fish species, including commercially important fish such as Atlantic cod (*Gadus morhua*), haddock (*Melanogrammus aeglefinus*), herring (*Clupea harengus*) and various species of demersal flatfish, Norwegian fjords are important spawning and nursing grounds. In fish, heavy metals such as Cd and Cu are of concern because it is easily accumulated and may disturb biological mechanisms even at relatively low, environmentally relevant exposure concentrations [5,7].

Intracellular Cu concentrations are tightly regulated in animals; however, excessive amounts may disturb protein function and induce oxidative stress [5,[8], [9], [10], [11]]. Cu toxicity varies between species, but early life stages are generally considered especially vulnerable [12]. Numerous physiological processes are affected by Cu in fish, including ion regulation (inhibition of Na^+^/K^+^-ATPase and subsequent disruption of osmoregulation), immunological function, metabolism and ultimately behaviour [5]. Cu is considered less toxic to fish in seawater compared to freshwater as free metal ions will to a larger degree be bound to complexes reducing the bioavailability, and seawater contains higher concentrations of competing ions such as calcium (Ca) [13]. However, little is known about the lethality and toxic impact of Cu on early life stages of marine fish species, but previous works reported Cu impacts on embryos and larvae of some species such as Atlantic herring (*Clupea harengus*), plaice (*Pleuronectes platessa*) and gilthead seabream (*Sparus aurata*) [14,15]. A recent study reported increased mortality, developmental and growth impacts and deformation in Cu exposed marine medaka (*Oryzias melastigma*) [16]. However, data on potential effects of Cu exposure on early life stages of commercially important species in the Northern Atlantic, such as Atlantic cod, remain scarce.

The aim of this study was to investigate whether Cu exposure at low, and thus environmentally relevant concentrations, affect the survival and development of Atlantic cod embryos and larvae. A dynamic energy budget (DEB) model, integrating developmental traits such as body size, yolk sac size, body weight and oxygen consumption, was applied to reveal potential impacts on energy allocation/availability under exposure and potential impacts on growth.

## 2 Material and methods

### 2.1 Experimental setup

Eggs of Atlantic cod (*Gadus morhua*) were obtained from a spawning brood stock of Atlantic cod at Austevoll Research Station (Institute of Marine Research). After fertilization, eggs were incubated for 24 h (6.2 °C, salinity 34.4‰) before being sent to SINTEF Sealab in cooled containers by air freight. Until the start of the exposure, eggs were kept in 50 L flow-through tanks supplied with filtered (1 μm) natural sea water from Trondheimsfjord (80 m; below thermocline; salinity 34‰; pH 7.6). Dead and unfertilized eggs were removed from the tank.

Exposure started at 4 days post fertilisation (3 dpf) and continued throughout the embryonic stage until 17 dpf, corresponding to 5 days post hatch (5 dph). Nominal Cu exposure concentrations were 0.5 μg L/L (low), 2 μg/L (medium), 6 μg/L (high). Natural seawater was used as negative controls (ctrl). The exposure concentrations were based on the guide for classification of environmental conditions in water from the Norwegian Environmental Agency [17]. The low and medium Cu concentration in this exposure corresponds to the class II/III - no effect to chronic effects from long-term exposure (0.3–2.6 μg/L), and high Cu concentration to class V – severe toxic effects (>5 μg/L).

Exposure solutions were prepared by dissolving CuSO_4_×5 H_2_O (Sigma-Aldrich, ACS reagent, ≥98 %) in ultrapure water (Milli-Q Plus, Millipore Corp.), which was subsequently diluted with filtered natural seawater to prepare seawater stock solutions. Exposure solutions were prepared by further diluting the seawater stocks. Exposures were performed in 1 L food grade polyethylene terephthalate (PETE) containers partly submerged in a flow through water from a temperature-controlled source (10 °C) water. The containers were thoroughly cleaned before use, rinsed with 1 M HNO_3_ and soaked with filtered seawater for 24 h. Before exposure onset, the containers were equilibrated for 24 h with respective Cu exposures concentrations to saturate container walls to reduce loss of Cu.

At 4 dpf approximately 500 eggs were added to each container and exposed until 5 dph. Each exposure was replicated (n = 7), whereof all replicates were used to determine hatching and mortality. Individuals from one replicate were used for daily determination of biometry and larval deformations as described below. To ensure sufficient oxygen saturation and slight mixing of the water, the exposure containers were supplied with air provided at low pumping rate with a tubing pump releasing single bubbles close to the bottom of the container. Oxygen saturation and temperature were measured daily using a phase fluorometer (NeoFox GT, Ocean Optics, Largo, US) equipped with a R-series FOSPOR electrode (Ocean Optics, Largo, US) and temperature compensated by a thermistor probe (NeoFox-TP, Ocean Optics, Largo, US). The ambient and water bath temperature was set to 10 °C with dimmed light conditions with a 12 h light/dark cycle. Larvae hatched before the main hatching event were removed from the exposure containers and kept under comparable exposure conditions in separate beakers. Similarly, unhatched eggs after the main hatching event were removed from the main exposure containers and kept separately. The exposure solutions were exchanged at 72 h of exposure, 144 h and at 2 dph, and at each time point approximately 2/3 of the water was exchanged.

### 2.2 Exposure verification and Cu uptake

Water samples were taken 4 times during the experiment, namely at the start (0 h), at 72 h, 144 h and at the end of the exposure, each before and after changing of the exposure solution, to determine dissolved Cu concentrations and eventual changes caused by exposure solution exchange. At each timepoint, 10 mL were taken from three exposure containers (n = 3) and filtered (0.45 μm, polyethersulfone, VWR International, USA). The vials were flushed with filtrate. The samples were acidified with 3 droplets of 50 % v/v nitric acid (HNO_3_, ultrapure grade, purified from HNO_3_ (AnalaR NORMAPUR®, VWR) in sub-boiling distillation system, Milestone, SubPur, Sorisole, BG, Italy), to achieve a final concentration of 0.1 M HNO_3_ and stored in dark at 4 °C prior to analysis. Copper concentrations were analyzed with ICP-HR-MS using a Thermo Finnigan model Element 2 instrument (Bremen, Germany) with a SC2 DX autosampler (with ULPA filter dust cover) and a PrepFAST flow injection analysis system (Elemental Scientific, Inc. Omaha, NE, USA). The instrument was calibrated with multielement standards. As blank samples, ultrapure water (MilliQ, Milestone) and sea water samples were analyzed together with the samples. The determined LOD for Cu in seawater was 0.6 μg/L.

Copper concentrations were further determined in eggs with developing embryos and hatched larvae. Before the start of the exposure, 6 replicates of 30 eggs (n = 30) were sampled to determine Cu concentrations before exposure onset. Further, samples (n = 30 eggs) were collected from 6 replicates of each exposure concentration at 96 h after the start of the exposure, while hatched larvae (n = 30 from 6 replicates per exposure) were sampled at 3 dph. The samples were kept frozen in polypropylene (PP) cryo-vials at −20 °C until preparation and analyses. After thawing, 1.5 mL of nitric acid (50 % v,v, Ultra-Pure grade, distillation with Milestone SubPur, Sorisole, BG, Italy) was added. The samples were digested in a heating chamber for 1 h at 60 °C, then for 2 h in 105 °C. After digestion, the samples were diluted with purified water to a final volume of 15 mL in metal free PP tubes. Final analyses were performed using an inductively coupled plasma triple quadrupole mass spectrometry instrument (ICPQQQ, Agilent 8800; Agilent Technologies, USA) with ^115^In and ^89^Y as internal standards (Inorganic Ventures, USA). As dry weight was not determined in the eggs and larvae collected for ICP-MS analyses, concentrations are expressed as ng/g individual, and/or were recalculated using average dry weights determined in other individuals (n = 78 for eggs; n = 60 for larvae). For Cu uptake in eggs and larvae we calculated the limit of quantification (LOQ) to 0.05 ng/egg or larvae (0.53 and 0.68 μg/g; dry mass).

### 2.3 Mortality and hatching

The number of eggs with dead embryos, hatched larvae, and dead larvae was assessed daily, and dead individuals and eggs shells of hatched larvae were removed. Hatched larvae were transferred to acid-washed glass beakers containing the same Cu exposure concentrations (ctrl, low, medium and high) and were held at the same temperature and light regime as the exposure containers. Larvae collected at different days were held separately and mortality in the groups was evaluated daily. Oxygen levels were assessed daily as described above, and the exposure solutions were partly exchanged to ensure comparable water quality to the main exposure containers.

### 2.4 Oxygen consumption

Oxygen consumption in both eggs with developing embryos as well as hatched larvae was measured in a micro-respirometer (Loligo® Microplate Respirometry System with MicroPlate™ software version 1.0.4 Loligo Systems, Denmark) with a volume of 940 μL per chamber. Two replicates per treatment were measured daily, each replicate containing six embryos or larvae. Mean oxygen consumption per individual was calculated based on the linear decline of oxygen concentration (minimum R^2^ -value of 0.9) corrected for temperature, air pressure and the number of individuals (n = 6) in the measurement chamber.

### 2.5 Dry weight

For each treatment, three replicates were sampled for dry weight daily. The eggs were quickly rinsed with distilled water, carefully blotted dry and placed in pre-weighted tin cups, with n = 3 eggs per cup. Larvae were quickly rinsed in distilled water, before being carefully transferred to pre-weighed tin cups in a drop of distilled water using a soft flat forceps, with n = 3 larvae per cup. The tin cups with eggs or larvae were dried for 48 h at 60 °C before weighing.

### 2.6 Deformation ranking and measurement of biometric traits

To determine potential impacts on development and growth and potential deformations, 6 larvae of each exposure group (ctrl, low, medium, high) were sampled daily, embedded in a methylcellulose gel, and imaged with a macroscope (model Z6APO, Leica Microsystems, Germany) using a CMOS camera (MC170HD, Leica Microsystems, Germany). Larvae were ranked (using anonymised numbers) for the occurrence of spine-, craniofacial-, jaw-, tail-, marginal finfold- and heart deformations, as well as alterations in pigmentation (lack of - or little pigmentation). The severity was ranked similarly to previous studies [18,19] using severity indices 0 (non), 1 (SI1, mild), 2 (SI2, moderate) to 3 (SI3, severe). Larvae imaged from all days were grouped for deformation analyses.

To determine impacts on larvae growth and development, biometric traits (standard length, myotome height, 2D body area, 2D yolk sac area and eye diameter) were automatically determined using the MASK-R CNN neural net architecture as decribed in more detail in ([20,21] submitted). Detected outliers were manually checked for accuracy of the automated process.

### 2.7 Statistics and data treatment

GraphPad Prism 7 (Graph-Pad Software Inc., USA) was used for data analyses. Data sets were analysed for normality (Shapiro-Wilk normality test) and analysed with either one-way ANOVA or Kruskal-Wallis tests and associated post-hoc tests to determine differences between groups.

To determine mortality of eggs/hatching success, we used an approach of Kotani et al. [22] to correct for sampled embryos. This approach assumes a constant mortality over the experimental duration and assigns a mortality rate to sampled individuals as well. We did, however, not use this approach to determine the overall mortality, as alive fish were selectively sampled, with the larvae sampled relatively late in the experiment which would potentially assign an unrealistic and biased mortality to the sampled cohort.

### 2.8 Dynamic energy budget (DEB)

The model used was the DEBkiss model for embryos, as explained in detail in Jager et al. [23]. In that paper, the model was parameterised and tested for Atlantic cod yolk-feeding stages using available data from the literature. This model can be used to integrate the responses of various traits to exposure. In this study, the data obtained from oxygen consumption measurements, dry-weight, and biometry measures (larvae only) were used to calibrate the model, and to evaluate potential effects of Cu exposure on the energy budget of exposed cod.

The projected 2D area (*A_y_*) and length of the yolk sac (*L_y_*) were used to calculate yolk volume (*V_y_*), using the relationship for a prolate spheroid as shown in equation 1:

$$V_{y}=\frac{8}{3\pi }\;\frac{A_{y}^{2}}{L_{y}}$$

Structural volume (*V_s_*) was estimated from the approximate area of the structural body (*A_s_*, total projected 2D area minus projected 2D yolk-sac area) and the myotome length (*L_s_*), assuming the relationship for a cylinder (equation 2):

$$V_{s}=\frac{\pi }{4}\;\frac{A_{s}^{2}}{L_{s}}$$

Other endpoints used for model fitting were standard length, total dry weight, total wet weight, and the oxygen consumption rate. In addition to the endpoints used for model calibration in the previous paper, we here used two additional endpoints, namely structural volume (post hatch only) and total wet weight (pre hatch only). The state variables in the model are the dry masses of yolk and structure. Structural volume was calculated from structural mass using the dry-weight density *d_V_*. Total wet weight was calculated from the yolk and structural volume, where yolk volume followed from yolk mass and the dry-weight density of yolk. For the pre-hatch live stages, the chorion weight was added.

Few model modifications were used to model these additional endpoints, relating to specific deviations from the model predictions. Since the embryo is metabolising yolk to fuel its maintenance and development, as evident from the use of oxygen, a decrease in dry weight is unavoidable. However, the total wet weight of the egg did not decrease over development. The logical conclusion is that the water content of the egg increases over development. It is possible that water is taken up in the egg to replace the biovolume of yolk that is burned. In the model, the wet weight was therefore kept constant over the egg stages at the value for *t* = 0. This is consistent with the increase in perivitelline space over the initial development of the egg [24], although the complete picture of the water relations in the developing egg is more complex. Similarly, when yolk runs out, the larvae must burn structural biomass, and hence shrink, to maintain their bodies and hence decrease in dry weight. Again, this was not observed in the data, and we decided to keep the modelled body volume constant upon shrinking, which also implies uptake of water to replace the burned biomass. Another modification was to use a slightly lower weight for the chorion: a rather arbitrary 0.01 mg instead of 0.02 mg used in the previous study. This was done as only very little decrease in dry weight was observed upon hatching in the current study. The chorion volume seems to be rather variable between and within individuals (see Jung et al. [25]), so it is conceivable that the current batch had a lower chorion weight than established previously.

We fitted the same parameters as in Jager et al. [23] and compared the results for the current data set to those established in that paper. With the additional measured endpoints wet weight and structural volume, we could also fit the dry-weight density *d_V_* (it was kept constant in [23], using reported values for cod larvae). The data set used for the previous calibration was for animals kept at 6 °C. In the current tests, temperature was slightly higher and increasing somewhat over time (8.8–10.3 °C during the experiment). We used the Arrhenius temperature correction for the rate constants in the model (equation 3):

$$F_{T}=\text{e}\text{x}\text{p}\left(\frac{T_{A}}{T_{ref}}-\frac{T_{A}}{T}\right)$$

Where *F_T_* is the factor by which the rate constants for assimilation and maintenance are multiplied, *T_A_* is the Arrhenius temperature for Atlantic cod (13500 K, as established in the add-my-pet project, see Marques et al. [26]), *T_ref_* is a reference temperature (taken as 6 °C to allow comparison to the previously established values), and *T* is the actual profile of measured temperatures in the test (interpolated with a cubic spline). All temperatures in this equation are entered in Kelvin.

Hatching time was set at 10.5 dpf, as most eggs hatched between 10 and 11 dpf in this study (see below). In the model, hatching time is only used for the dry-weight and wet-weight model output to simulate shedding of the chorion and the contents of the perivitelline space. The egg incubation temperature was around 9 °C, and using the Arrhenius relationship, that would imply a hatching time of 17.5 dpf for the reference temperature of 6 °C. This is quite consistent with the hatching time of approximately 16 days used in the previous study [23].

To test whether there is a dose-related effect of Cu, a stress factor *s* was added to the model, in DEBtox style ([27]; equation 4):

s=b max(0,c-z)

Where *c* is the average Cu concentration in water, *z* is a threshold, and *b* a linear effect strength. Toxicokinetics of Cu in yolk-feeding stages is potentially quite complex, and the observed effects (or lack thereof) certainly do not allow quantification of the time pattern of effect. Therefore, we used a constant stress factor here. This stress factor applied to either the assimilation or maintenance process in a linear fashion as in equations 5 and 6:

$$J_{Am}^{a}\to (1-s)J_{Am}^{a}$$

$$J_{M}^{v}\to (1+s)J_{M}^{v}$$

This allowed for statistical testing, to see whether adding the stress factor improves the fit, in which case, there would be a consistent dose-related effect of Cu on the measured traits.

The model was implemented in the BYOM platform under Matlab (see https://www.debtox.info/byom.html). The model was fitted using likelihood optimisation, where the likelihood was calculated assuming independent normal distributions both across time and across endpoints for the residuals after square-root transformation. Robust confidence intervals were derived by profiling of the likelihood function.

## 3 Results and discussion

### 3.1 Exposure conditions, Cu concentration validation and uptake

Temperature in the exposure containers varied slightly, with an average of 9.53 ± 0.54 °C (8.8–10.32 °C min– max). Oxygen levels remained satisfactory throughout the experiment, with typical oxygen levels in the exposure containers of 8.9 ± 0.1 ppm.

Average determined Cu concentrations were < LOD for ctrl (LOD of 0.6 μg/L), and 0.93 ± 0.19 μg/L, 3.54 ± 1.56 μg/L and 6.51 ± 1.74 μg/L for low, medium, and high exposure groups, respectively. Measured Cu concentrations were close to nominal exposure concentrations at the start of the experiment with < LOD in ctrl, and 0.82 ± 0.05, 2.2 ± 0.1 and 5.6 ± 0.18 μg/L in low, medium and high exposures, respectively. Concentrations increased slightly during the experiment and reached 1.2 ± 0.22, 1.3 ± 0.09, 5.16 ± 0.3 and 9.5 ± 3.4 μg/L at the last sampling point.

**Fig. 1:**
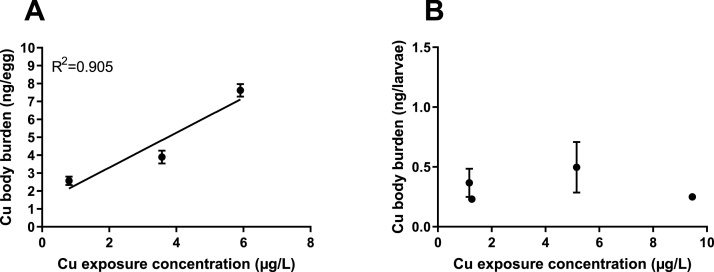
Cu exposure concentrations and relation to Cu body burden in eggs (A) and larvae (B). Cu concentrations in water are presented as those measured during the egg phase (A) or larvae phase (B). Data are presented as mean ± SE.

Results show that cod eggs accumulated Cu in an exposure dependent manner (Fig. 1A). Copper body burden (concentrations are shown as ng Cu per individual) is shown in Fig. 1. When recalculated for average dry weight, Cu concentrations in eggs sampled at the start of the experiment were 12.9 ± 3.7 μg/g dry weight. After exposure Cu concentrations were 25.7 ± 3.6, 27.4 ± 6.2, 41.6 ± 9.5 and 81.3 ± 9.2 μg/g dry weight in eggs of the ctrl, low, medium and high exposed groups, respectively. Copper concentrations were much lower in larvae, with 4.9 ± 3.9, 3.1 ± 0.4, 3.9 ± 1.2 and 3.4 ± 0.3 μg/g dry weight. Further, Cu body burden of eggs was correlated to the Cu concentrations in exposure dispersion (R^2^ = 0.917; Fig. 1A), but this was not observed in larvae, where Cu body burden was found to be unrelated to Cu exposure concentrations (R^2^ = 0.004; Fig. 1B). This is in agreement with a recent study investigating effects of Cu exposure on early life stages of marine medaka, reporting that medaka eggs contained more Cu compared to larvae, with 90 % of the Cu accumulated on the chorion [16]. Copper concentrations in the eggs from the negative control group in this study were comparable to those published by Wang et al. [16], who reported up to 13 μg Cu per g dry mass. In contrast, Cu concentrations in exposed eggs and larvae in this study were lower compared to those reported by Wang et al. [16], however, exposure concentrations used here were also lower. The results may suggest that, additionally to Cu being associated with the chorion during embryogenesis, larvae are potentially able to physiologically regulate Cu uptake/excretion from waterborne exposure.

### 3.2 Hatching, and mortality of developing embryos and hatched larvae

**Fig. 2:**
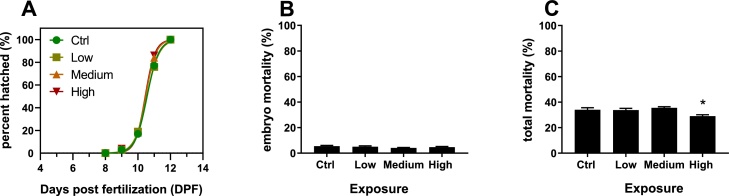
Hatching time of cod exposed to no (ctrl), low, medium and high Cu concentrations (A). Mortality presented as percent of the population of Atlantic cod embryos (B) and total individuals (embryos and larvae) in ctrl groups and following Cu exposures (C). Data are presented as mean (A) and mean ± SE (B,C). Significant differences (p < 0.05) are indicated (*).

Hatching time was relatively similar between exposure groups, with high and medium exposure groups hatching marginally earlier than ctrl and low groups (Fig. 2A). Most larvae hatched between 10 and 12 dpf, with 50 % of the larvae hatched at 10.56 dpf (95 % CI 10.5–10.63) in ctrl, 10.55 dpf (95 % CI 10.51–10.6) in low exposures, 10.48 dpf (95 % CI 10.44–10.51) in medium exposures and 10.45 dpf (95 % CI 10.42–10.49) in high exposure groups (Fig. 2A). The percentage of unhatched eggs with embryos at 13 dpf was below 0.5 % in all groups and was thus regarded negligible. Early hatching was reported for Atlantic herring eggs exposed to Cu [14]. Wang et al. [16] reported that medaka embryos exposed to between 20–80 μg/L had a hatching peak one day before the control group, while higher exposures (160–320 μg/L) caused a 2-day hatching delay. The authors further reported significant effects of Cu on hatching success, however only at concentrations of ≥160 μg/L, which is significantly higher than the highest exposure concentrations applied in this study.

Despite the Cu accumulation in the eggs with the developing embryos, we did not observe effects on survival at the tested concentrations, exposure timepoints (exposure start at 4 dpf) and environmental conditions (temperature and light) as exposure to Cu did not affect mortality of the developing embryos (Fig. 2A). For the study over all embryo mortality in the eggs was generally low; 5.5 ± 0.5 % for ctrl, and 5.0 ± 0.7, 4.1 ± 0.3 and 4.7 ± 0.5 % for low, medium and high exposures, respectively. Similarly, total mortality (eggs with embryos and larvae) was not elevated in exposure groups, but was even significantly lower in the highest exposure group (Fig. 2B, p = 0.0286), potentially due to a decrease in overall bacterial activity as Cu is used as antibacterial, antifungal and general antiparasitic treatment for both freshwater and seawater fish [28]. To our knowledge, effects of Cu exposure on Atlantic cod eggs with developing embryos and hatched larvae have not been reported yet, but Granmo et al. [29] studied effects of combined exposures of Cu and triazine on Atlantic cod early life stages, reporting a decreased survival (although not significant) in the highest exposure concentration of 11.5 μg/L Cu in combination with 5 μg/L triazine [29]. Studies on early life stages of other marine fish species show that Cu exposure causes increased mortality in both eggs with developing embryos and larvae, however, reported effect concentrations for survival are usually above 50 μg/L [15,16,30]. It is further reported that some the sensitivity varies between life stages and developments events [16]. Reinardy et al. [31] reported increased mortality in cod larvae, but not in embryos following exposure to mine tailings from a copper mine. However, dissolved/bioavailable Cu concentrations were, with 3.4 μg/L not higher than in ctrl exposures with 4.3 μg/L (measured with ICP-IOS) [31]. The authors further report Cu accumulation in the chorion of embryos, but not in larvae, which is agreement to our study [31].

### 3.3 Deformations

**Fig. 3:**
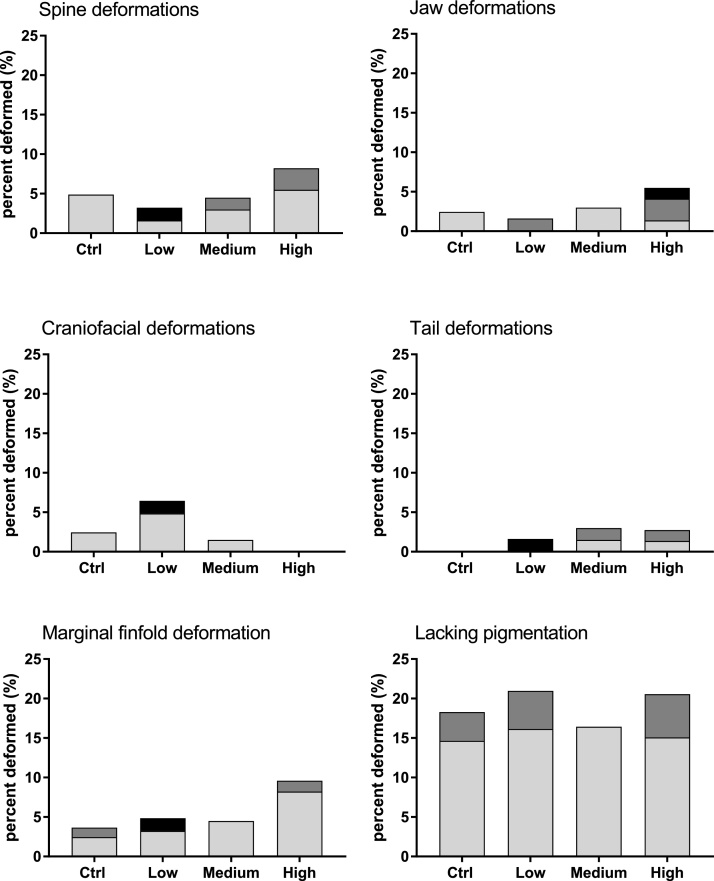
Relative amount of larvae (% of imaged individuals) with deformations in ctrl and Cu exposed groups. Different colours are displayed for mild (light grey), moderate (dark grey) and severe (black) deformations.

Exposure to Cu did not induce a significant increase in deformations in larvae of Atlantic cod in this study. In the ctrl group, 18.3 % of all imaged larvae had some deformation or had low or lacking pigmentation. This was similar for larvae in the Cu exposed groups, with percentage of larvae that had 1 or more deformations being 21, 16.4 and 20.5 % in low, medium and high exposures, respectively. The relative amounts of the most frequently detected deformations are shown in Fig. 3. Limited information exists on the occurrence of such deformations in natural populations of Atlantic cod, but differences in morphology (body shape) has been reported between distinct populations and during rearing at different temperatures [32]. High occurrence of vertebral deformities is a challenge in the cod aquaculture industry, and vertebral malformations have been observed in 1–24 % and 3–34 % of larvae from wild and farmed broodstocks, respectively [33]. Many skeletal deformations have onset during late-embryonic and early larval stages when chondrogenic and osteogenic differentiation occurs and may impact survival and behaviour later in life. Deformations, especially skeletal deformations, are a known effect of heavy metal exposure of fish larvae [34]. Copper was shown to induce malformations such as reduced pigmentation (in African sharptooth catfish; *Clarias gariepinus*), spinal deformation (in Japanese medaka, Oryzias latipes) or curved tails (in goldfish, Carassius auratus) in exposed eggs with developing embryos [[35], [36], [37]], however, most studies were conducted on freshwater species. Blaxter and co-authors reported deformations of herring larvae when exposed to 30 μg/L or 90 μg/L of Cu immediately after fertilisation, with no such effects observed when the exposure started later, at 4 dpf [14]. Wang et al. [16] reported morphological abnormalities in marine medaka when exposed to Cu above 160 μg/L.

### 3.4 Effects on development and energy budget

**Fig. 4:**
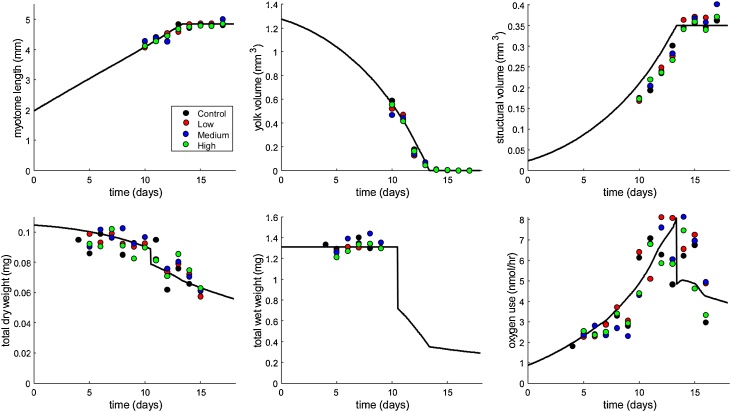
Simultaneous fit to the data for various life-history traits. Plotted points are means for the different treatments (ctrl in black, low in red, medium in blue and high in green). Model parameters are provided in Table 1.

Integration of the determined biometric traits and oxygen consumption showed that the measurements fitted well to the applied DEBkiss model (Fig. 4). Although the structural volume was slightly overestimated by the model, potentially related to our assumption of constant water content for yolk and structure throughout development, while the reality is more complex [24]. The fitted model parameters in this study are quite similar to the values established previously using literature data [23]. These results support the biometrical measurements from the image analysis, and the usefulness of the model to explain the growth and development of the yolk-feeding stages of cod.

**Table 1:** List of parameters of the DEBkiss model estimated in this study. Other parameters were kept constant to the values established previously for Atlantic cod [23]. All parameters referenced to 6 °C, using an Arrhenius temperature of 13500 K. Confidence intervals are 95 % likelihood-based intervals.

Symbol	Explanation	Jager et al. [23]	This study	Unit
	**Primary parameters**			
$J_{Am}^{a}$	Max. area-specific assimilation rate	16.0 (14.7−17.1)	15.0 (14.1−15.9)	μg/mm^2^/d^1^
$J_{M}^{v}$	Volume-specific maintenance costs	4.37 (3.87−5.02)	3.29 (2.99−3.60)	μg/mm^3^/d^1^
κ	Fraction of assimilation flux for soma	1 (0.949−1)	1 (0.995−1)	(-)

	**Initial states**			
$W_{B0}$	Initial yolk buffer in egg	100 (96.9−104)	90.0 (88.6−91.7)	μg
$W_{V0}$	Initial structure in egg	2.35 (1.48−3.64)	4.58 (3.24−6.07)	μg

	**Conversions**			
$d_{B}$	Dry-weight density of yolk buffer	0.0745 (0.0714−0.0796)	0.0706 (0.0692−0.0722)	mg/mm^3^
$d_{V}$	Dry-weight density of structure	0.15 (n.e.)	0.194 (0.188−0.199)	mg/mm^3^
$\delta_{M}$	Shape-correction coefficient	0.157 (0.151−0.162)	0.145 (0.144−0.146)	(-)

	**Toxicity parameters**			
b	Effect strength	not used	not identifiable	L/μg
z	Threshold for effects	not used	not identifiable	μg/L

Adding the stress factor to the model did not improve the fit at all; the optimised log likelihood is same with and without the stress function (in fitting, the toxicity parameters *b* and *z* of equation 4 converge to values that produce *s* = 0). This supports the visual observation that there is no Cu stress on the determined growth traits followed in this study at the tested Cu concentrations.

In contrast, effects of Cu exposure on developmental and growth-related measures such as body length and yolk sac size have been reported as sensitive endpoints in previous studies [16]. However, differences can potentially be related to exposure concentrations, as the concentrations tested in the current study were below concentrations reported to cause significant development effects in marine embryos and larvae [14,16].

## 4 Conclusions

Our results show that Cu is accumulated in an exposure concentration dependent manner in eggs with developing embryos of Atlantic cod, but not in hatched larvae. Furthermore, the exposure concentrations tested (<10 μg/L) did not cause increased mortality, deformations or impacts on growth, development or energy budget in embryos and larvae upon exposure onset at 4 dpf to exposure termination at 5 dph. While acute and sublethal effects in the literature are reported for higher Cu concentrations (>50 μg/L) in early life stages of marine fish, it is noteworthy that lower effect concentrations are described for embryos exposed immediately after fertilisation, or larvae at later live stages.

## Author statement

Julia Farkas: Conceptualization, Validation, Investigation, Formal analyses, Writing original draft, Writing review and editing, Visualization, Supervision, Project administration, Funding acquisition. Linn H. Svendheim: Validation, Investigation, Formal analyses. Tjalling Jager: Conceptualization, Methodology, Formal analyses, Writing original draft. Tomasz M. Ciesielski: Investigation, Formal analyses, Writing review and editing. Trond Nordtug: Methodology, Investigation. Bjarne Kvæstad: Methodology, Software. Bjørn H. Hansen: Conceptualization, Investigation, Supervision, Writing review and editing. Torstein Kristensen: Conceptualization, Writing review and editing. Dag Altin: Investigation, Writing review and editing. Pål A. Olsvik: Project administration, Funding acquisition, Supervision, Conceptualization, Writing original draft, Writing review and editing.

## Conflict of interest

The authors declare no conflict of interest.

## Data availability

No data was used for the research described in the article.

Data will be made available on request.

All data is within the manuscript and figure.

## Declaration of Competing Interest

The authors report no declarations of interest.
